# Exploring Nursing Students’ Experiences in a Brief Virtual Reality–Enhanced Workshop: Cross-Sectional Exploratory Study

**DOI:** 10.2196/85780

**Published:** 2026-04-02

**Authors:** Niklas Ferdinand Carlsson, Leonie Klompstra, Mats Westas

**Affiliations:** 1 University Library, Linköping University, Bredgatan 33, Norrköping, 60221, Sweden, 46 0700896482; 2 Department of Health, Medicine and Caring Sciences (HMV), Linköping University, Norrköping, Sweden

**Keywords:** virtual reality, education, community of inquiry, cross-sectional studies, experiential learning, education 4.0, academic library makerspace, nursing

## Abstract

**Background:**

There is limited evidence on how brief, optional virtual reality (VR) experiences can be used with first-semester nursing students as experiential learning strategies to support understanding of foundational nursing concepts, outside of mandatory coursework or full-scale simulations. Additionally, little is known about students’ and teachers’ perceptions of VR as a low-stakes, supplemental learning strategy introduced early in nursing education. Examining these experiences can provide insight into the pedagogical value and scalability of VR-enhanced learning within the formal nursing curriculum.

**Objective:**

This study explored students’ and teachers’ experiences of a brief, optional, VR-enhanced workshop offered outside mandatory coursework in first-semester nursing education and described students’ perceptions of cognitive, social, and teaching presence.

**Methods:**

This was a cross-sectional evaluation at a Swedish public university. A single-session workshop, co-designed by nursing teachers and the university library makerspace (implementation context), combined brief headset exposures (sympathetic arousal via a short roller coaster experience and parasympathetic engagement via a short guided meditation), peer vital-sign practice (instructional aid), small-group synthesis, and a guided debrief aligned with the community of inquiry (CoI) framework. Immediately after the session, students completed a demographics questionnaire, a 7-item workshop-specific VR-perception set, and the 34-item CoI instrument, plus 2 open-ended items; teachers provided short reflections. Analyses were descriptive for quantitative data and summative content analysis of open-ended responses. Participants included 11.9% (16/134) of the invited first-semester students (mean age 25 years, SD 5.1; 15/16, 93.8% women; 6/16, 37.5% with prior VR exposure) and 3 teachers.

**Results:**

Most students agreed or strongly agreed that VR enhanced analysis and observation (12/16, 75%), exploration of phenomena (14/16, 87.5%), conceptual understanding and engagement (13/16, 81.3%), teacher support (13/16, 81.3%), and relevance to the session (14/16, 87.5%). CoI ratings indicated moderately positive perceptions (total mean 3.36, SD 0.44 on a 5-point scale), with cognitive presence rated the highest (mean 3.48, SD 0.41) and exploration being the top subdomain (mean 4.48, SD 0.49); design and organization and facilitation were similar (mean 3.42, SD 0.55 each), whereas direct instruction was rated lower (mean 2.88, SD 0.92). Open-ended remarks described links between theory and embodied experience and noted practical challenges.

**Conclusions:**

This study used an early, optional format; the results showed that brief, contrastive VR exposures paired with scaffolded inquiry and a guided debrief were perceived as pedagogically valuable for exploring foundational physiological concepts, while also highlighting feasibility and logistical considerations for routine teaching. Findings are preliminary and reflect session-level perceptions from a small, self-selected sample; nevertheless, they suggest that structured, low-stakes VR may serve as a feasible supplemental strategy in first-semester nursing education, with implications for potential scalability.

## Introduction

### Background

Contemporary educational paradigms increasingly emphasize the need for immersive, interactive learning environments that integrate theoretical instruction with practical, hands-on experiences, grounded in educational theory and supported by institutional pedagogical frameworks [1-4]. In undergraduate nursing, where foundational concepts about physiology and patient care must be internalized early, there is a growing interest in introducing virtual reality (VR) early in the curriculum as an optional, low-stakes complement to core coursework to spark engagement and support conceptual understanding in the first semester [5].

A growing evidence base suggests that VR can enhance learning by providing immersive, repeatable, and safe experiences that bridge abstract concepts and embodied understanding. Meta-analyses in nursing education have reported positive effects on theoretical knowledge, technical skills, self-confidence, and learner satisfaction when VR is integrated with attention to design features and instructional support [6-8]. At the same time, findings are not uniform: outcomes vary by intervention design and outcome type, and some studies note that while presence increases, learning can suffer when cognitive load is high or scaffolding is insufficient [5,9,10]. Practical considerations, such as cybersickness risks influenced by exposure duration, content, and locomotion, also warrant attention when working with first-time users [11].

There is limited evidence on how brief, optional VR experiences can be used with first-semester nursing students as experiential learning strategies to support understanding of foundational nursing concepts, outside of mandatory coursework or full-scale simulations. Additionally, little is known about students’ and teachers’ perceptions of VR as a low-stakes, supplemental learning strategy introduced early in nursing education. Examining these experiences can provide important insight into the pedagogical value and scalability of VR-enhanced learning within the formal nursing curriculum. Reports frequently emphasize usability or novelty yet underdescribe how short VR exposures are pedagogically integrated to connect conceptual content (eg, autonomic physiology) with immediate experiences and collaborative sensemaking [5,10].

To guide a purposefully designed, concise activity, we drew on education 4.0 (learner-centered, experience-driven, and digitally supported learning); constructive alignment (coherence among intended learning outcomes, teaching and learning activities, and assessment); and the community of inquiry (CoI) framework (cognitive, social, and teaching presence) [12-18]. Framing VR as one tool within an aligned pedagogical design helps ensure that brief exposures are structured to foster inquiry, link embodied responses to concepts, and support facilitated reflection.

To address these gaps, we implemented and evaluated a brief, optional, VR-enhanced workshop offered outside mandatory coursework in first-semester nursing education. The model combined short headset exposures designed to elicit contrasting autonomic responses, peer practice of vital-sign measurement to support observation and discussion, small-group synthesis, and a guided debrief aligned to the CoI practical inquiry cycle. We describe the implementation and examine students’ and teachers’ experiences, focusing on students’ perceptions of cognitive, social, and teaching presence as indicators of the perceived learning environment [15-17].

### Objective

This study explores students’ and teachers’ experiences of a brief, optional VR-enhanced workshop offered outside mandatory coursework in first-semester nursing education and described students’ perceptions of cognitive, social, and teaching presence.

## Methods

### Design and Reporting

This cross-sectional study evaluated an optional, VR-enhanced workshop using postworkshop surveys (Likert-type items and open-ended responses). No behavioral observation checklists were used. Reporting follows STROBE (Strengthening the Reporting of Observational Studies in Epidemiology) guidance for cross-sectional studies [19].

### Setting, Participants, and Recruitment

The activity took place at a Swedish public university (south central region). All first-semester nursing students (N=134) were invited to participate via announcements on the online classroom platform and brief in-class invitations. Registration remained open for one week, and 16 (11.9%) students who expressed interest received a confirmation message securing their place in the workshop. Participation was voluntary and outside mandatory coursework. In addition, 3 nursing teachers participated across all workshop phases and provided brief reflections. Library makerspace staff co-facilitated implementation (roles are detailed in subsequent sections) but were not included in the teacher feedback items.

### Workshop

This optional, supplemental, VR-enhanced experiential learning activity (distinct from a clinical simulation scenario) was offered alongside the first-semester curriculum and was not included in required assessments or mandatory components. Students were divided into 2 groups of 8 for workflow and logistical reasons (nonrandom allocation). Each group completed one VR module, either a short roller-coaster exposure (sympathetic arousal) or a short guided meditation (parasympathetic engagement), followed by inquiry tasks and a guided debrief (Table 1). The workshop was implemented as an optional addition to existing teaching (ie, not part of the school’s required simulation infrastructure) to explore perceived experiences with a concise, aligned model.

**Table 1.** Time structure of the workshop.

Component of the workshop	Time (min)
Virtual reality exposure and peer vital-sign practice	60
Working on answering the given questions and preparing a PowerPoint presentation	75
Group presentations and reflection	45

### Implementation Roles (Contextual)

The workshop was co-designed and co-facilitated by 2 units: nursing teachers (pedagogical design, alignment with first-semester concepts, and facilitation of the debrief and links to physiology) and university library makerspace staff (readiness and comfort protocols for first-time VR use; headset onboarding; hygiene, sanitation, and logistics; and facilitation of information-seeking and source evaluation during the inquiry task). This cross-unit collaboration supported delivery; no evaluation of collaboration effects (eg, feasibility, process outcomes, or comparative effectiveness) was undertaken, and collaboration was not a study outcome.

### VR Exposures and Rationale

We selected brief headset exposures to contrast autonomic activation states: (1) sympathetic activation via an approximately 4-minute Epic Roller Coasters track and (2) parasympathetic engagement via an approximately 5- to 6-minute TRIPP guided meditation, primarily to support conceptual understanding of autonomic physiology and secondarily to maintain comfort and accessibility for first-time users [20,21]. Evidence summarized in prior reviews indicates that cybersickness risk is associated with exposure duration, visual stimulation, and locomotion and content; comfort-oriented, shorter sessions are commonly recommended for first-time users [11,22].

### Vital-Sign Practice Procedure

Before headset use, each student had resting baseline values recorded (heart rate, blood pressure, respiratory rate, oxygen saturation, and tympanic temperature) using standard training instruments in the student practice ward. To minimize movement artifact and measurement reactivity, spot-checks were obtained immediately after the VR exposure (not during). These measurements were intended to support observations, understanding of sympathetic and parasympathetic responses, and postexperience discussion, rather than research-grade physiological inference; no continuous monitoring was performed, and readings were not designed to isolate specific organ-system mechanisms.

### Grouping and Sequence

Two groups of 8 proceeded in parallel (roller coaster *or* meditation). Assigning 1 module per group supported the single-session timebox, comfort for first-time users, and a contrastive learning design that allowed cross-group comparison during synthesis and debrief. Students could reference their own baseline and immediate postexposure readings (eg, heart rate, blood pressure, and respiratory rate) in slides and discussion to illustrate sympathetic vs parasympathetic patterns.

### Inquiry Tasks and Presentation (CoI Aligned)

Small groups completed 3 tasks designed to link embodied responses to conceptual understanding and to support the CoI practical inquiry cycle [15,16].

First, explain observations with evidence—locate and use credible sources (textbooks, peer-reviewed literature, and institutional resources) to interpret observed changes consistent with sympathetic vs parasympathetic mechanisms.

Second, synthesize a brief minilesson—prepare a short slide deck that links observations and theory (autonomic physiology; cardiovascular regulation), applies source appraisal, and communicates clearly to peers. Students could optionally include their own heart rate, blood pressure, and respiratory rate comparisons; content on heart dynamics, signaling pathways, and energy metabolism was addressed via literature-based synthesis.

Third, present and debrief (guided)—deliver the minilesson and then participate in a guided debrief aligned with CoI prompts (eg, triggering event, exploration and integration, and bounded resolution). Facilitation emphasized guided reflection and feedback tailored to the goals of the workshop.

### Scaffolds: Prompts and Rubrics

Instructors used structured supports to scaffold the activity. The observation log rubric included baseline vs immediate postexposure readings and salient sensations, along with a brief plain-language interpretation. The source evaluation rubric included authority, accuracy, relevance, and timeliness. The synthesis rubric included claim evidence reasoning, alignment to the learning goal, and clarity for peers. These scaffolds guided observation, evidence appraisal, and coherent explanation and were not graded assessments.

The source evaluation rubric included authority, accuracy, relevance, and timeliness. The synthesis rubric included claim evidence reasoning, alignment to the learning goal, and clarity for peers. These scaffolds guided observation, evidence appraisal, and coherent explanation and were not graded assessments.

### Data Collection and Measures (Combined)

Students completed an anonymous postworkshop survey with 4 components.

First, the survey collected demographic data on age, gender, and prior VR headset experience.

Second, the survey included 7 VR-perception items that were workshop-specific and targeted perceived analysis and exploration, conceptual understanding and engagement, teacher support, relevance, and the value of learning new technology. These items were tailored to this workshop and were not intended as a validated general instrument; therefore, reliability statistics were not reported for this subscale.

Third, the CoI instrument (34 items) was used and its wording was adapted minimally to the in-person, single-session context (eg, replacing “course” with “workshop activity”) while preserving full coverage of the constructs (cognitive, social, and teaching presence). Given the small sample, we did not estimate reliability in this study; we relied on prior validation literature [15,16].

Fourth, the survey included 2 open-ended questions addressing challenges, barriers, and opportunities for learning with VR, including reflections that could reference sensations experienced. Teachers (nursing teachers) provided brief reflections, whereas makerspace staff co-facilitated implementation but did not complete teacher items.

### Ethical Considerations

Participation was voluntary with written informed consent obtained from all participants. Responses were anonymized to protect privacy and confidentiality. Formal ethics review was not required for this type of educational research at our institution and no sensitive personal data were collected [23]. No compensation was provided to participants.

### Analysis

Quantitative data were summarized descriptively (no inferential statistics were performed). The 7-item workshop-specific perceptions were reported as distributions; no reliability statistics were calculated. For the CoI instrument, we reported subscale and total scores descriptively and relied on prior validation literature rather than estimating reliability in this small sample. Open-ended responses underwent summative content analysis (2 coders independently open coded the data and reconciled differences through discussion; categories were not assumed to be mutually exclusive) [24].

## Results

### Overview of Outcomes

**Figure 1.**
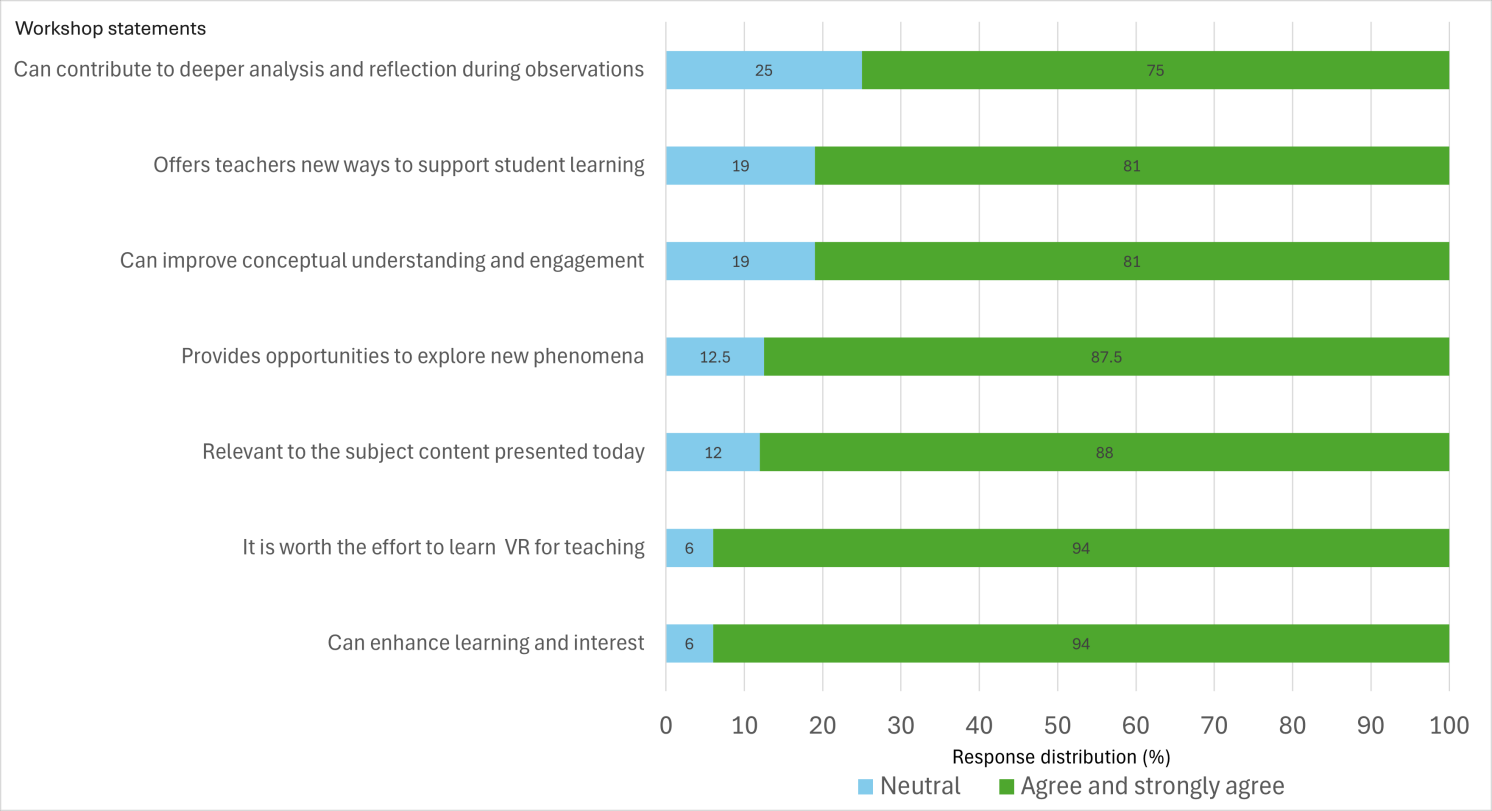
Nursing student ratings concerning using virtual reality (VR) in education.

Table 2 summarizes students’ CoI scores (teaching, social, and cognitive presence and their subdomains); Figure 1 shows agree and  strongly agree distributions for the 7-item VR-perception statements, sorted from highest to lowest.

**Table 2.** Mean score of the community of inquiry (CoI) survey instrument (n=16).

CoI survey elements	Scores, mean (SD)
Teaching presence	3.28 (0.57)
Design and organization	3.42 (0.55)
Facilitation	3.42 (0.55)
Direct instruction	2.88 (0.92)
Social presence	3.26 (0.60)
Affective expression	3.04 (0.65)
Open communication	3.35 (0.68)
Group cohesion	3.38 (0.69)
Cognitive presence	3.48 (0.41)
Triggering event	3.58 (0.46)
Exploration	4.48 (0.49)
Integration	3.46 (0.46)
Resolution	3.42 (0.49)
Total score	3.36 (0.44)

Of the students at the nursing school (n=134), 16 (11.9%) signed up to participate. The study included 16 students (mean age 25, SD 5.1; range 20‐38 years), of whom 15 (93.8%) were women. Of the 16 students, 37.5% (n=6) had prior experience using VR headsets. Among these, 2 (12.5%) students had used VR headsets for gaming purposes and 3 (18.8%) students experienced motion sickness or stress during the use of VR. One (6.3%) student did not provide details about their prior experiences with VR. In addition to the students, 3 teachers (mean age 51; SD 10.0, range 43‐62 years; all male) participated in the workshop. Two teachers had previous experience with VR headsets: one had used them on a few occasions and the other had explored both recreational and pedagogical applications of VR.

Of the 16 students, most agreed or strongly agreed that the VR headsets could increase analysis and observation (n=12, 75%), could help with exploring a new phenomenon (n=14, 87.5%), could increase concept knowledge and engagement (n=13, 81.3%), could create new possibilities for teachers to support their students in learning (n=13, 81.3%), and were relevant to use for the topic of the workshop (n=14, 87.5%) and thought it was worth learning a new technology (n=15, 93.8%; Figure 1).

### CoI Results

For interpretability, teaching presence reflects design and organization, facilitation, and direct instruction; social presence reflects affective expression, open communication, and group cohesion; and cognitive presence reflects triggering event, exploration, integration, and resolution. Participants’ ratings indicated moderately positive perceptions of the workshop’s learning environment (Table 2). Within teaching presence, design and organization and facilitation were similar (mean 3.42, SD 0.55 each), whereas direct instruction was lower (mean 2.88,  SD 0.92). Social presence averaged 3.26 (SD 0.60), with subdomains ranging from affective expression (mean 3.04,  SD 0.65) to group cohesion (mean 3.38, SD 0.69). Cognitive presence had the highest overall mean 3.48 (SD 0.41), with exploration rated highest among its subdomains (mean 4.48, SD 0.49), followed by triggering event (mean 3.58,  SD 0.46), integration (mean 3.46, SD 0.46), and resolution (mean 3.42,  SD 0.49). Students referenced their own baseline and immediate postexposure readings (eg, heart rate, blood pressure, and respiratory rate) descriptively to illustrate sympathetic versus parasympathetic patterns; these readings were not analyzed as outcomes.

### Qualitative Results (Open-Ended Responses)

#### Overview

As outlined in the Methods section (summative content analysis), we analyzed anonymous, session-specific open-ended comments. A total of 15 (93.8%) of the 16 students and all 3 teachers provided responses (18/19, 94.7% contributors in total). The unit of analysis was the individual comment. Codes were merged into 3 descriptive categories, and the counts reported represent the number of unique contributors per category. Categories were not mutually exclusive, and the quotations included are illustrative rather than exhaustive [24].

#### Challenges to VR Use

Participants (9/18, 50%) noted cybersickness and physiological discomfort, headset comfort and time burden, access, logistical, and technical issues, and unfamiliarity and the learning curve.

What I can think of is that one can easily become nauseous, and that leads to difficulty concentrating.

[Participant 2402]

Overall experience can be time-consuming.

[Participant 2403]

Technical issues. Digital literacy is required in the teacher competency.

[Participant 2419]

Everyone is not familiar with the technology.

[Participant 2415]

#### Opportunities for Learning

Students and teachers (16/18, 88.9%) highlighted visualization and immersion to aid understanding and recall, stronger links between theory and observed autonomic responses, low-stakes practice, and a structured debrief that supported collaborative sensemaking.

I could experience the connection between theory and practice.

[Participant 2415]

It allows for your senses to be used in other ways with VR which allows for learning in new ways.

[Participant 2407]

To experience something and the symptoms that arise with it makes it easier to remember in the learning process.

[Participant 2416]

To mix learning methods gives you a greater understanding and a wider picture.

[Participant 2411]

#### Feasibility and Logistics

Practical considerations according to participants (5/18, 27.8%) included equipment availability, onboarding, hygiene, sanitation, and fit adjustments, occasional software and hardware restarts, and scheduling.

Technical aspects need to work, both software and hardware.

[Participant 2418]

Students need guidance to find the right levels where they do not get caught in technical details.

[Participant 2417]

There might be a need to educate the students in VR-basics.

[Participant 2406]

Immediate sensations (eg, nausea, dizziness, and stress) were self-reported by students during the guided debrief and/or the open-ended survey; vital-sign values were not displayed in-headset.

#### Interpretation

These brief comments help contextualize the session-level experiences but do not provide evidence of longer-term learning or engagement. The counts are descriptive, and no claims of thematic saturation are made, consistent with STROBE guidance for transparent reporting in cross-sectional studies [19].

#### Link to Cognitive Presence

The qualitative category “opportunities for learning” (16/18, 88.9%) aligns with several components of cognitive presence. Students’ noticing was primarily subjective during exposure and later referenced to postexposure spot-check readings in the debrief, which together reflect CoI triggering events. Their emphasis on visualization, immersion, and linking theory to physiological responses corresponds to exploration, which also received the highest quantitative subdomain score (mean 4.48,  SD 0.49). References to small-group synthesis activities and the guided debrief illustrate integration, as students worked to make sense of information across sources. Comments referring to feasibility constraints and the brief scope of the workshop help explain why resolution was rated lower than exploration (mean 3.42, SD 0.49 vs mean 4.48,  SD 0.49), although it remained moderate overall. Together, the open-ended responses complement and contextualize the quantitative profile of cognitive presence while remaining descriptive and limited to session-level experiences.

### Instructor Observations

Instructors noted that the format enabled students to observe VR-elicited physiological responses and link these observations to autonomic physiology concepts during the group debrief. These impressions are limited to the session; given the brief, individual VR exposure and the supplemental nature of the workshop, the study does not provide evidence for sustained changes in active or reflective learning or overall engagement. Terms such as “personal” or “meaningful” engagement were not measured. Peer vital-sign measurements were used to facilitate discussion and illustrate concepts (not to evaluate learning outcomes).

## Discussion

### Principal Findings

This study explored students’ and teachers’ experiences with a brief, optional, VR-enhanced workshop introduced outside mandatory coursework in first-semester nursing education. Students perceived the activity as pedagogically valuable for linking autonomic physiology to embodied experience and for exploration within the learning environment. Interpreted through the CoI framework, the overall profile suggested moderately positive perceptions, with the strongest pattern in cognitive presence: exploration. That pattern is consistent with short, inquiry-driven designs that prompt noticing, questioning, and sensemaking during and after exposure [15,16]. In doing so, this study addresses the identified gap by showing how a brief, optional, low-stakes VR exposure can be positioned early in the curriculum and by documenting students’ and teachers’ perceptions of its pedagogical value and perceived feasibility, with implications for potential scalability as a supplemental strategy. These results reflect session-level perceptions during the workshop’s collaborative elements (peer readings, small-group synthesis and presentations, and guided debrief) and should be interpreted cautiously given the brief, optional, single-session design and small sample (n=16); the study did not assess longer-term changes in engagement or learning.

### What the Findings Contribute

A central contribution of this work is the demonstration of a low-stakes, early-semester format in which students experience contrasting autonomic states themselves via a short roller-coaster exposure (sympathetic arousal) and a short guided meditation (parasympathetic engagement) and then interpret those experiences through peer discussion, literature-informed minilessons, and a guided debrief. This sequence appears well-suited to catalyzing curiosity (CoI “triggering event” to subsequent “exploration”) and to making abstract physiological concepts more tangible early in the curriculum [15,16]. The optional nature and the brief duration likely reduced performance pressure while preserving focus on conceptual understanding and engagement, which helps explain the salience of exploration even in a single session. Students’ noticing was primarily subjective during exposure (eg, feelings of arousal or relaxation) and was later referenced to postexposure spot-check readings during the guided debrief and minilessons, providing concrete anchors for interpretation.

Positioned against the broader VR literature, these session-level perceptions align with findings that VR can enhance learning, particularly motivation, satisfaction, knowledge, and skills, when instructional design and scaffolding are explicit [5-8,25]. At the same time, they are consistent with evidence that presence alone does not guarantee learning and that insufficient scaffolding can increase cognitive load and dampen outcomes [5,9]. Our brief, contrastive exposure format with a structured debrief addresses that design dependence by pairing immersive moments with meaning-making activities rather than treating VR as a standalone novelty.

### Interpretation in Context (Education 4.0, Constructive Alignment, and CoI)

The workshop design drew on education 4.0 as a high-level orientation to learner-centered, experience-driven, and digitally supported learning, and on constructive alignment to ensure coherence among intended learning outcomes, teaching and learning activities, and reflection [12-14,18]. Notably, vital sign values were not displayed in-headset; students’ “triggering events” arose from subjective sensations during exposure and subsequent reference to postexposure spot checks in the debrief. In practical terms, alignment was enacted by contrastive exposures that instantiate target concepts (autonomic arousal vs relaxation), scaffolded inquiry that connects observations to theory (minilessons with source appraisal), and a brief guided debrief aligned with CoI (cognitive, social, and teaching presence) to support sensemaking and closure [15,16]. The study did not conduct competency assessments and does not claim competence gains; rather, it offers exploratory evidence that a concise, optional format can be perceived as pedagogically valuable and feasible for early supplemental use.

### Implementation Considerations

Students’ reflections and facilitator notes highlighted pragmatic factors important for routine teaching: headset fit and hygiene protocols, onboarding time for first-time users, and occasional software and hardware restarts. Such start-up frictions are common when integrating immersive devices and should be planned for in scheduling and staffing [5]. The brief contrastive design also served a comfort and accessibility function (limited headset time and seated options), which is advisable given that cybersickness risk is associated with exposure duration, visual stimulation, and locomotion; carefully shorter, comfort-oriented sessions are appropriate for novices [11,22].

### Implications and Future Recommendations

The findings of this study suggest several practical implications and directions for future research. Positioning early integration of VR as an optional, low-stakes supplement may be beneficial. Offering brief, clearly scaffolded experiences in the first semester may help to prime curiosity and anchor foundational physiology in embodied experience [5,6].

In addition, using contrastive exposures to make mechanistic contrasts vivid (eg, sympathetic vs parasympathetic) and following these with a guided debrief that traces CoI’s practical inquiry cycle from triggering events to bounded resolution may support learning [15,16].

Scaffolding with simple tools (observation logs and source evaluation and synthesis rubrics) allows students to produce evidence-informed explanations without adding grading burden [12,13].

Planning for logistics and comfort includes onboarding, sanitation, fit checks, and rapid troubleshooting, and exposures should be kept brief for first-time users [11,22].

Research next steps include moving beyond session-level perceptions to comparative and mixed methods designs that examine knowledge retention, reflective writing quality, and observational rubrics, as well as assessing accessibility (nonheadset alternatives) and scalability (equipment and time models) across cohorts [7,9,25].

### Limitations

This study has several limitations that should be considered when interpreting the findings. First, the activity was optional, single-session, and conducted in a single institution with a small, self-selected sample (n=16), which limits generalizability and precludes inference about longer-term effects. Second, outcomes relied on postsession self-report (VR-perception items and the CoI instrument) without objective educational performance measures, comparators, or follow-up; findings therefore reflect session-level perceptions rather than effectiveness or competence.

Third, vital sign readings (eg, heart rate, blood pressure, and respiratory rate) were collected only as instructional aids to support observation and discussion. In this study, we recorded a resting baseline before exposure and spot-checks immediately after exposure; we did not collect data during exposure or conduct continuous physiological monitoring, and values were obtained with standard training instruments by peers rather than research-grade devices. Consequently, these readings are not suitable for physiological inference, characterization of within-session dynamics, or clean separation of phenomena such as anticipatory arousal. Immediate sensations (eg, nausea, dizziness, and stress) were self-reported during the guided debrief and/or in open-ended survey responses and were not systematically assessed with a validated instrument.

Fourth, the 7-item VR-perception set was tailored to this workshop and was not intended as a validated, generalizable scale; therefore, we do not report reliability for this set. For the CoI instrument, we adapted wording minimally to fit an in-person, single-session context, but given the small sample, we did not estimate reliability in this study and instead relied on prior validation evidence reported elsewhere. Finally, equipment and logistical factors and cross-unit collaboration supported implementation but were not evaluated as outcomes.

Taken together, these factors indicate that results should be interpreted as exploratory evidence about perceived learning environment and feasibility for an early, optional, low-stakes VR activity; they do not demonstrate objective learning gains or competency development. Directions for addressing these constraints (eg, comparative designs, larger samples, objective outcomes, structured measures of discomfort, and continuous physiological monitoring) are outlined in the Implications and Future Recommendations section.

### Conclusions

A brief optional VR workshop introduced early in nursing education can provide low-stakes, embodied experiences that students perceive as helpful for exploring foundational physiological concepts. When paired with scaffolded inquiry and a guided debrief aligned with CoI, short exposures can prompt curiosity and sensemaking within the formal curriculum, offering a practical, scalable supplement to the required coursework [5,15].

## Abbreviations

CoI: community of inquiry
STROBE: Strengthening the Reporting of Observational Studies in Epidemiology
VR: virtual reality
